# Energetic stability states in mast cell activation syndrome: operationalizing reserve, pressure, and threshold collapse

**DOI:** 10.3389/fimmu.2026.1865449

**Published:** 2026-07-13

**Authors:** Richard Tellier

**Affiliations:** Independent Researcher, Land O’ Lakes, FL, United States

**Keywords:** crash susceptibility, diagnostic stratification, energetic stability states, mast cell activation syndrome (MCAS), multisystem synchronization, nonlinear dynamics, perturbation–recovery dynamics, pressure–reserve dynamics

## Abstract

Mast cell activation syndrome (MCAS) is characterized by pronounced heterogeneity, fluctuating severity, delayed post-perturbation crashes analogous to those documented in post-exertional malaise, and highly variable clinical trajectories that are poorly explained by mediator-centric diagnostic approaches. Patients with similar symptom profiles or laboratory findings often follow divergent courses, while resting biomarkers frequently fail to correlate with functional capacity or prognosis. Beyond serving as markers of activation, mast-cell mediators influence neural, vascular, epithelial, immune, and autonomic systems, providing a biologically plausible mechanism through which local perturbations may propagate into multisystem instability. Building on a pressure–reserve framework of energetic constraint, this paper proposes a stability-state classification framework for interpreting system behavior and identifying operating regimes. Four operational stability classifications are proposed—recovery-capable, plateau, slow drift, and crash-prone—based on the relationship between energetic reserve and reactive pressure, modulated by the degree of multisystem synchronization. Recovery-capable, plateau, and slow-drift classifications are conceptual regions along a continuous fragility axis, whereas crash-prone behavior is proposed as a threshold-crossing regime characterized by impaired recovery dynamics, hysteresis, and increased susceptibility to instability. Three complementary stability axes are introduced: energetic reserve, dominant pressure domain (dominant ingress), and synchronization. Together, these axes generate characteristic classification signatures that remain observable despite overlapping mediator profiles and fluctuating baseline values. This framework reframes MCAS as a disorder of state-dependent instability rather than mediator burden alone, provides a structured means to interpret heterogeneity, stratify patients prognostically, and track trajectory over time, and offers a foundation for longitudinal monitoring and future validation studies grounded in system dynamics rather than isolated biomarkers.

## Graphical Abstract

**Figure:**
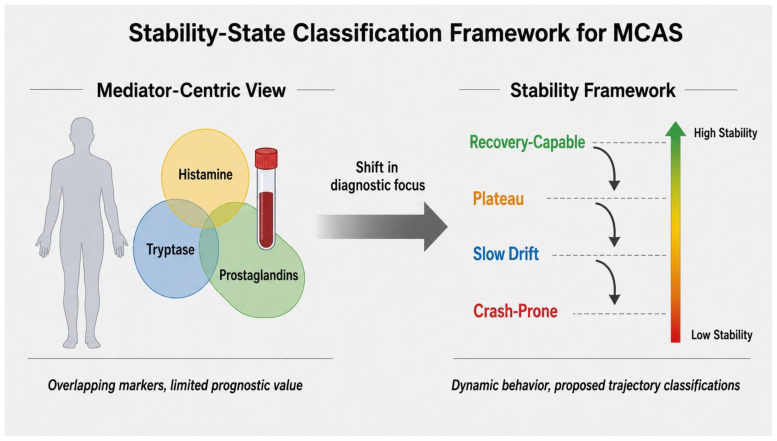

## 1 Introduction

Mast Cell Activation Syndrome (MCAS) is characterized by marked clinical heterogeneity, fluctuating severity, and unpredictable trajectories poorly explained by existing diagnostic frameworks (1, 2). Patients with similar mediator elevations may follow divergent courses—from gradual recovery to progressive instability with episodic multisystem collapse—while individuals with severe functional impairment may demonstrate minimal laboratory abnormalities at rest (3). These observations suggest that MCAS severity is not determined solely by mediator burden, but by broader system-level dynamics governing energetic reserve, reactive pressure, and failure propagation (4–10).

Current diagnostic frameworks remain largely mediator-centric, emphasizing histamine, tryptase, and prostaglandins (7, 8, 11). While these measures confirm mast cell involvement, they provide limited insight into why symptoms vary over time, why exertion or stress provokes delayed post-perturbation crashes—analogous to post-exertional malaise documented in related conditions (12–14)—in some patients but not others, or why treatments reducing mediator release may stabilize some individuals while offering little benefit to others (5–8, 10). These recurring observations—mediator–symptom mismatch, delayed crashes, and heterogeneous trajectories—are better understood as emergent properties of system-level instability than as isolated mediator effects.

MCAS provides a particularly informative model for examining stability behavior because mast-cell mediators function not only as markers of activation but also as active contributors to physiologic pressure. Histamine, prostaglandins, leukotrienes, cytokines, and proteases influence neural, vascular, epithelial, immune, and autonomic systems simultaneously (15). Through these effects, mast-cell activation may amplify physiologic load across multiple domains and contribute to failure propagation and multisystem synchronization. While the stability dynamics described in this framework may extend beyond MCAS, the syndrome provides a clinically relevant model in which pressure accumulation, network amplification, and instability are frequently observed together (7, 9–11).

Recent systems-oriented models of physiologic instability and critical transitions motivate reframing MCAS as a disorder of sentinel-cell instability emphasizing energetic vulnerability, nonlinear threshold behavior, and network amplification across immune, neural, vascular, and barrier systems (16–22). However, while such models clarify why instability persists and propagates, a critical gap remains: how to recognize, classify, and longitudinally track these dynamic operating regimes. Existing clinical tools largely capture static snapshots rather than stability regimes, offering limited capacity to distinguish individuals operating with substantial reserve from those approaching instability thresholds or exhibiting recurrent crash-prone behavior. As a result, prognosis remains uncertain, longitudinal change is difficult to interpret, and clinically meaningful differences in recovery dynamics often go unrecognized (23–25).

This paper addresses that gap by proposing a stability-state classification framework for MCAS grounded in pressure–reserve dynamics. The framework classifies patients according to their position along a stability continuum, identifies the dominant domains through which reactive pressure is expressed, and characterizes the degree to which failures synchronize across sentinel systems. Four operational classifications are proposed—recovery-capable, plateau, slow drift, and crash-prone—with crash-prone behavior representing a hypothesized threshold-crossing regime distinguished by altered recovery dynamics and instability under perturbation (20–22).

The goal is not to redefine MCAS as a new disease entity or to propose treatment algorithms, but to introduce a stability-state language and classification structure capable of explaining heterogeneity, forecasting instability, and enabling rational stratification for research and longitudinal monitoring.

Importantly, this framework differs from existing constructs such as allostatic load or disease severity staging. Allostatic load and resilience indices quantify cumulative burden using static or aggregated markers. In contrast, the present framework focuses on dynamic system behavior, emphasizing the relationship between energetic reserve, reactive pressure, and cross-system synchronization. Two individuals with similar allostatic load may occupy fundamentally different operating regimes; the framework’s primary unit of analysis is not cumulative damage, but operating regime and state transition (16, 17, 19). In this framework, the primary distinction is not between four presumed biological categories, but between regions of a continuous fragility spectrum and the potential emergence of threshold behavior associated with crash-prone instability.

## 2 Conceptual framework

### 2.1 Core constructs and operational axes

Stability in MCAS cannot be inferred from mediator levels alone, as mediator release reflects downstream expression rather than upstream capacity. Stability emerges from the interaction of three relational constructs:

Energetic reserve refers to the capacity of the system to maintain functional energetic gradients—mitochondrial membrane potential, ATP availability, redox balance—under demand and to recover predictably following perturbation (20–22, 26–28). Reserve is a dynamic property inferred from recovery kinetics, rebound completeness, tolerance to stress, and presence or absence of hysteresis, not from resting measurements. A system may appear normal at rest yet possess limited capacity to absorb additional load. Operationally, reserve is assessed through perturbation–recovery behavior: candidate readouts include mitochondrial membrane potential stability, ATP reserve proxies, NAD^+^/NADH-related redox balance, and lactate–pyruvate dynamics (20–22, 26–29). Key indicators are rebound speed (time to return toward baseline), completeness (extent to which baseline function is re-established), and hysteresis (persistence of dysfunction after load removal).

Reactive pressure refers to the aggregate energetic and signaling load imposed on the system from internal and external sources, defined relationally by its impact on reserve and system stability independent of specific etiology (16, 19–22). The same pressure may be well tolerated in a high-reserve system while producing instability in a low-reserve system. Multiple low-grade inputs may combine to exceed reserve even when no single factor appears dominant.

In MCAS, mast-cell mediators may contribute directly to reactive pressure by influencing neural, vascular, epithelial, immune, and autonomic systems simultaneously. In this context, mediator release is considered not only a marker of activation but also a potential contributor to pressure accumulation and failure propagation across interconnected systems (7, 9–11).

For operational measurement, reactive pressure is treated as current cumulative load rather than symptom severity, trigger sensitivity, or recovery impairment. This distinction is important because symptom response and recovery behavior belong primarily to the reserve construct, whereas reactive pressure reflects the active internal and external sources of load acting upon the system. Candidate sources of pressure include environmental exposures, dietary challenges, sleep disruption, physical exertion, emotional stress, illness, inflammation, and other concurrent physiologic demands.

Dominant ingress designates the primary domain through which reactive pressure most consistently predicts instability at a given time—assigned when a domain reproducibly precedes or predicts instability across repeated perturbations rather than merely being present (20–22). Pressure domains include excitability/Ca²^+^-linked pressure, immune-derived pressure, barrier/endotoxin-linked pressure, hypoxia–reperfusion or metabolic mismatch, and mitochondrial-intrinsic amplification. Dominant ingress is assigned when a single domain reproducibly precedes instability across multiple perturbation–recovery cycles and demonstrates greater predictive value than competing domains. Assignment is provisional and may shift over time as underlying pressure sources change. When two or more domains demonstrate comparable predictive strength, no dominant ingress is assigned and the profile is designated mixed. Prospective validation will be required to establish the reproducibility and temporal stability of ingress assignment.

Synchronization refers to the degree to which dysfunction across sentinel systems occurs in temporal alignment following perturbation. High synchronization converts otherwise tolerable disturbances into systemic instability by aligning energetic stress across immune, neural, vascular, metabolic, and barrier systems (18, 26, 30, 31). Low synchronization allows perturbations to remain compartmentalized. Synchronization is determined by timing and co-occurrence across systems, not by the magnitude of dysfunction within any single domain. Operationally, it is inferred from temporal clustering of symptoms across domains (gastrointestinal, neurologic, vascular, dermatologic) within defined time windows and from parallel shifts in physiologic signals (heart rate variability, blood pressure, temperature, sleep) (18, 29–31). Synchronization is treated here as an observable property of system behavior rather than a specific biological mechanism. Low synchronization reflects temporally compartmentalized disturbances; high synchronization reflects tightly coupled, high-amplitude temporal clustering constituting network-level instability.

**Figure 1:**
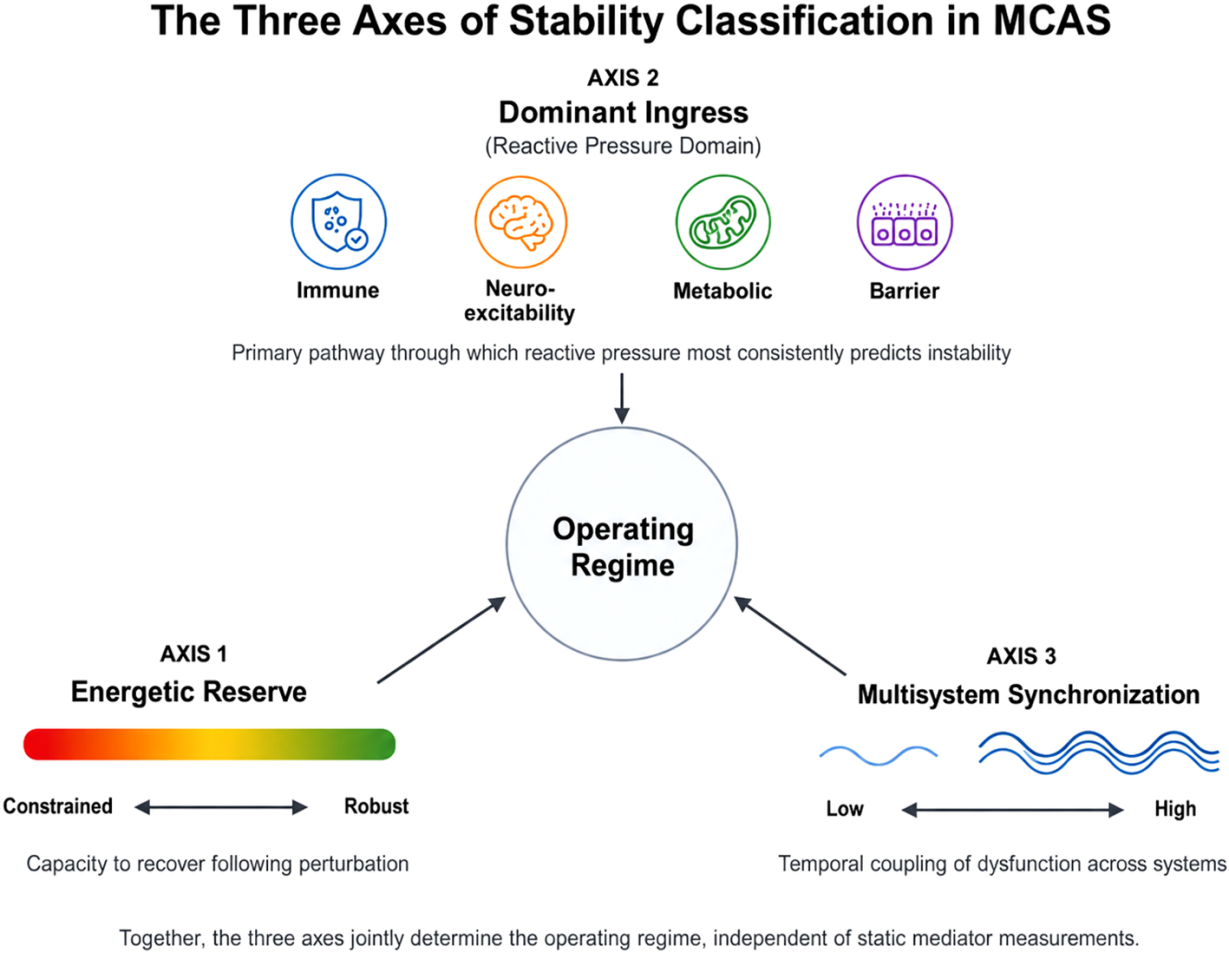
The three axes of stability classification in MCAS. Triangular schematic illustrating the three axes that jointly characterize position within the proposed stability classification framework. Axis 1 (Energetic Reserve): gradient from constrained to robust reserve, reflecting the system’s capacity to maintain energetic function and recover following perturbation. Axis 2 (Dominant Ingress/Reactive Pressure Domain): representative domains (immune, neuro-excitability, metabolic, barrier) denoting the primary pathway through which reactive pressure most consistently predicts instability. Axis 3 (Multisystem Synchronization): gradient from low to high synchronization, reflecting the degree of temporal coupling of dysfunction across sentinel systems. Together, these axes characterize system operating regime independent of static mediator measurements.

Together, these three constructs define the axes of a stability-classification coordinate system for MCAS (**Figure 1**).

### 2.2 Mast-cell contributions to reserve and the mediator pressure source

The dynamics described above—reserve, pressure, and synchronization—are general properties of multisystem regulation and are not, in themselves, specific to MCAS. What distinguishes MCAS within this framework is the nature of its dominant pressure source. In most sentinel populations, the cell acts primarily as a transducer of pressure: positioned near a functional threshold, it propagates instability when load converges upon it. The mast cell occupies an additional role. Through regulated and recurrent mediator release, it is also a generator of pressure—injecting histamine, prostaglandins, leukotrienes, proteases, and cytokines that act on neural, vascular, epithelial, immune, and autonomic systems (7, 11, 32). In MCAS, therefore, a portion of the reactive pressure the system must absorb is endogenous and arises from within the network itself.

This source has a self-amplifying character. Several mediator effects can, in turn, lower the threshold for further mast-cell activation—for example, through neuropeptide and neurogenic signaling at mast-cell–nerve interfaces and through autonomic and vascular changes that accompany instability (33, 34). To the extent that such return pathways operate, mediator release constitutes a feed-forward pressure source: activation raises systemic load, and the resulting instability can provoke further activation. We describe this as feed-forward rather than strictly recursive, since the strength and consistency of the return pathways remain to be established; characterizing them is an explicit aim of validation.

Mast-cell activation is also linked to the reserve side of the framework. Calcium flux, membrane-potential dynamics, and reactive oxygen species generated during activation couple mediator release to mitochondrial membrane-potential and redox strain, providing a cellular route by which repeated activation can draw down energetic reserve without necessarily producing overt resting abnormality (35). In this view, mast-cell activity contributes to both sides of the pressure–reserve relation in MCAS: it adds endogenous, potentially feed-forward pressure, and it can erode the reserve available to buffer it. This dual contribution offers a biologically grounded reason why MCAS so frequently exhibits pressure accumulation, network amplification, and instability together, while leaving the underlying stability dynamics applicable, in principle, to other multisystem conditions whose dominant pressure sources differ.

### 2.3 Stability as an operating regime

These constructs give rise to recurring patterns of system behavior that can be organized into operational stability classifications. Within a pressure–reserve framework, stability is determined not by symptom burden or mediator levels alone, but by the relationship between energetic reserve, reactive pressure, and synchronization under perturbation (20–22).

Importantly, the present framework does not require the existence of four discrete biological states. Rather, it proposes a continuous fragility spectrum along which reserve, pressure, and synchronization vary over time. Recovery-capable, plateau, and slow-drift classifications are best understood as operational regions along this spectrum, reflecting progressively reduced stability margin and increasing vulnerability to perturbation. Individuals may move between these regions gradually, without requiring a distinct biological transition (20–22, 36–38).

A separate possibility is that crash-prone behavior represents a threshold-crossing regime characterized by altered recovery dynamics. In this model, instability is no longer defined solely by the magnitude of symptoms or pressure load, but by changes in system behavior following perturbation. Candidate features of this regime include prolonged recovery, hysteresis, increasing synchronization, and failure to reliably re-establish baseline function after stress (20–22).

Accordingly, four operational classifications are proposed:

#### 2.3.1 Recovery-capable

Energetic reserve exceeds reactive pressure. Perturbations are absorbed and recovery is predictable, with minimal hysteresis and limited failure propagation.

#### 2.3.2 Plateau

Reserve approximately matches pressure. The system remains stable but operates near its functional margin, demonstrating a constrained ceiling and slower recovery.

#### 2.3.3 Slow drift

Pressure chronically and slightly exceeds reserve. Stability margin gradually erodes over time, resulting in progressively prolonged recovery and increasing sensitivity to routine demands.

#### 2.3.4 Crash-prone

Pressure episodically overwhelms severely constrained reserve, producing recurrent instability characterized by impaired recovery, high synchronization, and proximity to functional thresholds. Within the present framework, crash-prone behavior is treated as a candidate threshold-crossing regime requiring prospective validation.

**Figure 2:**
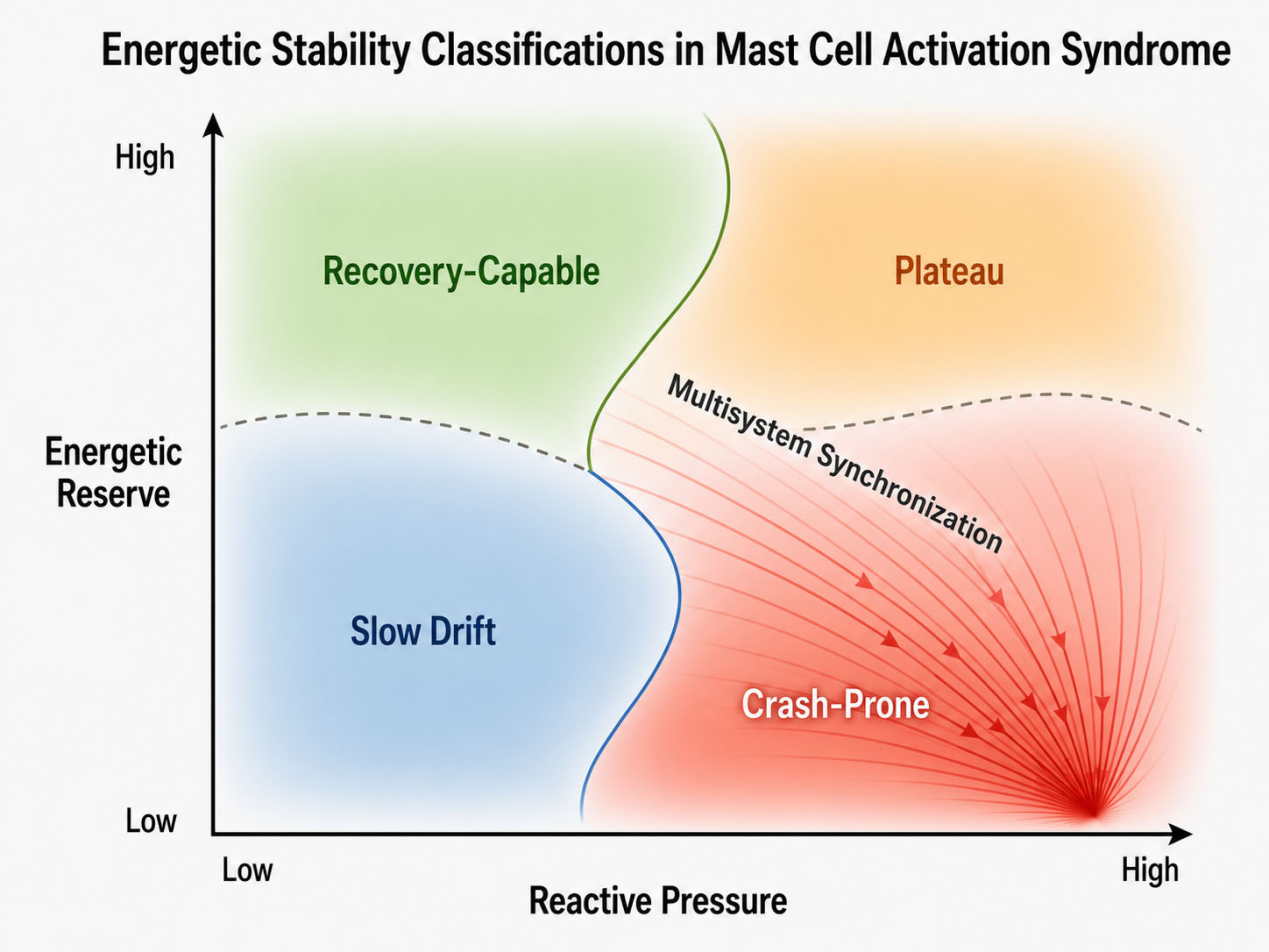
Energetic stability classifications in mast cell activation syndrome. Conceptual state-space diagram illustrating the proposed stability classifications as a function of energetic reserve (vertical axis) and reactive pressure (horizontal axis). Recovery-capable classifications occupy the high-reserve, low-pressure region. Plateau classifications represent a compensated high-pressure state in which reserve remains sufficient to prevent collapse but adaptive ceiling is constrained. Slow-drift classifications arise where pressure chronically and slightly exceeds reserve, leading to progressive erosion of stability margin. Crash-prone behavior, modulated by high multisystem synchronization (shown by radiating lines), occupies the low-reserve, high-pressure region and is proposed as a candidate threshold-crossing regime associated with altered recovery dynamics. Boundaries between regions are conceptual and do not represent empirically validated diagnostic cutoffs. Individuals may transition between classifications over time.

The spatial relationship between reserve, pressure, and synchronization across these classifications is illustrated in **Figure 2**.

## 3 Stability axes and classification signatures

### 3.1 Classification-specific signatures

The three axes generate observable classification signatures across the proposed stability classifications. Different combinations of reserve, dominant ingress, and synchronization produce distinguishable patterns despite overlapping mediator profiles. These classifications are intended as operational descriptors of system behavior rather than fixed biological categories. **Table 1** summarizes the distinguishing features; illustrative clinical patterns are provided in Section 4.5 (20–22, 36–38).

**Table 1:** Proposed stability classifications in MCAS: hypothetical conceptual characteristics and dynamic features.

Stability classification	Pressure–reserve relationship	Baseline pattern	Response to perturbation	Recovery kinetics	Synchronization
Recovery-Capable	Pressure < Reserve	Intermittent, mild symptoms; preserved function	Transient, localized	Rapid, complete; minimal hysteresis	Low; compartmentalized
Plateau	Pressure ≈ Reserve	Stable but constrained; limited ceiling	Predictable flares at limits	Slow but consistent	Moderate; low-amplitude clustering
Slow Drift	Pressure > Reserve (chronic, slight)	Progressive symptom expansion	Growing sensitivity to routine demands	Progressively prolonged	Increasing; expanding involvement
Crash-Prone	Pressure episodically overwhelms reserve	Apparent stability punctuated by crashes	Disproportionate, delayed, multisystem collapse	Chaotic, incomplete; marked hysteresis	High; tight temporal clustering

The classifications presented here constitute a hypothetical conceptual model and are not validated diagnostic categories; quantitative definitions and empirical validation are required before they can be applied diagnostically. Classification derives from dynamic behavior—reserve, recovery kinetics, and synchronization—rather than mediator levels or symptom severity measured at rest. Recovery-capable, plateau, and slow-drift classifications represent operational regions along a continuous fragility spectrum. Crash-prone behavior is proposed as a candidate threshold-crossing regime characterized by altered recovery dynamics and increased susceptibility to instability.

**Figure 3:**
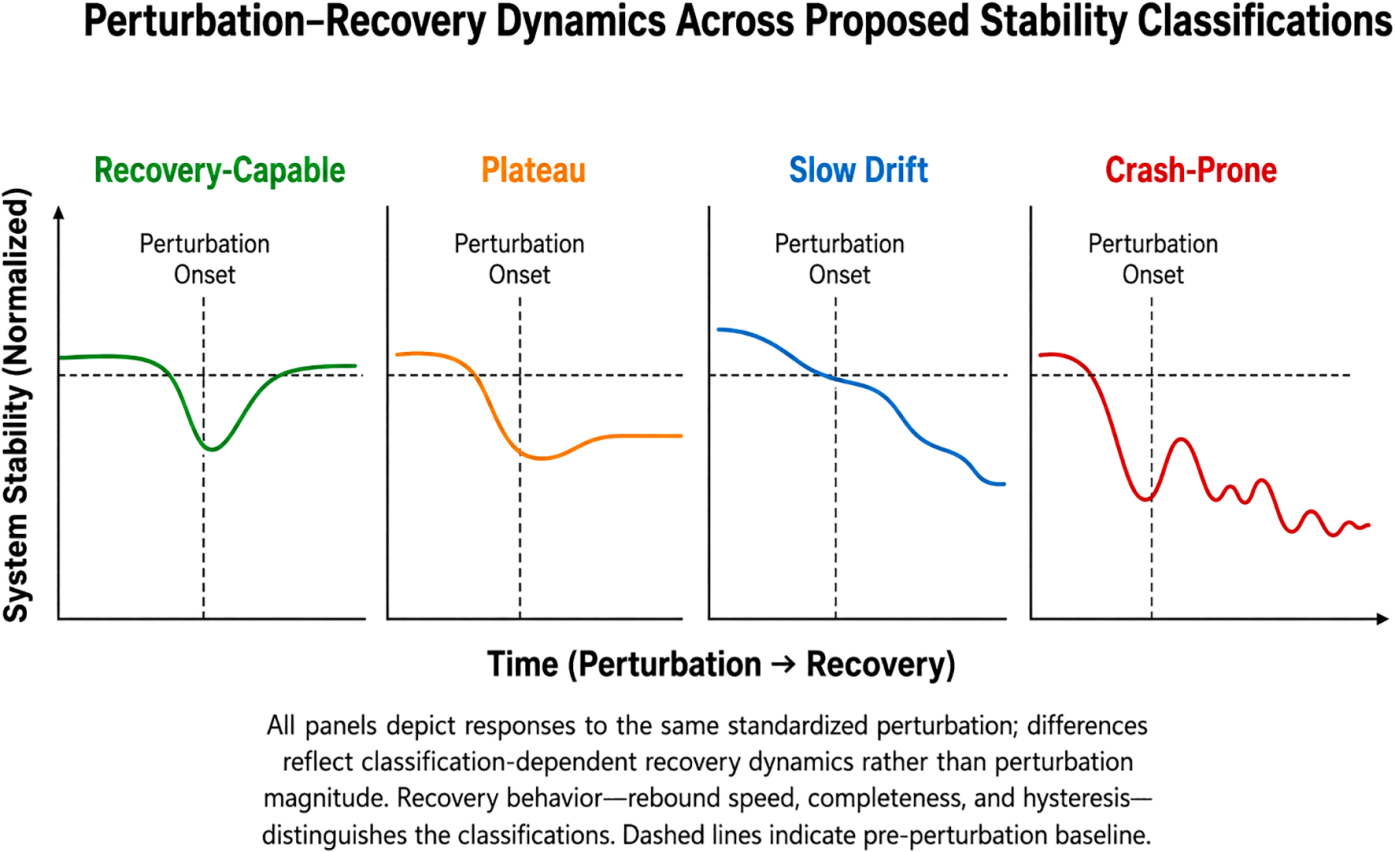
Perturbation–recovery dynamics across proposed stability classifications. Four-panel time-series plot showing normalized system stability over time following a standardized perturbation onset, indicated by the vertical dashed line. All panels depict responses to the same standardized perturbation; differences reflect classification-dependent recovery dynamics rather than perturbation magnitude. Dashed horizontal lines indicate the pre-perturbation baseline. Recovery-capable (green): rapid, complete rebound with minimal hysteresis. Plateau (orange): delayed but consistent recovery with reduced reserve margin. Slow drift (blue): progressively prolonged recovery with gradual erosion of baseline stability over the recovery interval. Crash-prone (red): prolonged, nonlinear recovery characterized by marked hysteresis, instability, paradoxical worsening after attempts to increase throughput, and incomplete restoration of baseline stability.

Across all classifications, mediator levels may overlap substantially and fluctuate independently of functional status. What differentiates classifications is not the presence of specific markers, but how the system behaves under load—how perturbations are absorbed, how recovery unfolds, and how dysfunction synchronizes across domains. Recovery kinetics across the proposed classifications following a standardized perturbation are illustrated in **Figure 3**.

### 3.2 Operationalization through the state classification instrument

To support reproducibility and prospective validation, the three proposed stability axes can be operationalized through a structured State Classification Instrument (SCI). The research version of the SCI is designed to operationalize three hypothesized stability axes: Reserve Deficit, Reactive Pressure, and Synchronization. Reserve Deficit captures impaired recovery kinetics, delayed worsening, incomplete rebound, and day-to-day variability in recovery capacity. Reactive Pressure captures current cumulative load from ongoing internal and external stressors, including environmental exposures, dietary load, sleep disruption, physical demand, emotional stress, illness, inflammation, hormonal or digestive load, sensory load, and simultaneous stressors. Synchronization captures multisystem temporal coupling, including co-occurrence, propagation, and coordinated symptom episodes. Reserve Deficit operationalizes the reserve construct described in §2.1, Reactive Pressure operationalizes current cumulative load acting on the system, and Synchronization operationalizes the degree of temporal coupling across sentinel systems.

The expanded research form is not intended to assign a stability classification at the point of administration. Instead, it provides item-level and axis-level data for validation (**Table 2**). Item-level psychometric analyses, including exploratory and confirmatory factor analysis, are used first to evaluate whether individual items load onto their intended constructs and whether a reduced short-form instrument can be derived. Only after this measurement structure is established are axis scores used to test whether participants cluster into reproducible stability classifications. The existence, number, and boundaries of any resulting classifications are treated as empirical questions rather than assumptions. This ensures that any classification structure reflects empirical system behavior rather than a predefined scoring rule, and that stability-state assignment remains a research outcome pending prospective validation.

**Table 2:** Proposed stability classifications linked to expected clinical manifestations, candidate physiologic markers, and validation endpoints.

Classification	Expected clinical manifestations	Candidate physiologic markers	Validation endpoint
Recovery-capable	Transient, localized symptoms; preserved function	Rapid HRV recovery; prompt lactate clearance; minimal hysteresis	Stable SCI profile; longitudinal monitoring confirms above-threshold trajectory
Plateau	Stable but constrained; predictable flares at limits	Slowed but consistent recovery; modest lactate persistence	SCI axis clustering with perturbation testing demonstrating reduced margin without collapse
Slow drift	Progressive symptom expansion; rising sensitivity to routine demands	Progressively prolonged recovery; altered lactate/lactate–pyruvate dynamics	Repeated SCI administration demonstrating directional change; recovery-vs-collapse trajectory analysis
Crash-prone	Apparent stability punctuated by delayed multisystem collapse	Chaotic, incomplete recovery; large hysteresis; paradoxical response to load	Dense time-series analysis; SCI profile associated with threshold-crossing behavior and hysteresis on challenge

This is presented as an illustrative operationalization rather than a finalized instrument. Specific numeric cut-points are deliberately not asserted; establishing and calibrating thresholds—including whether a crash boundary is identifiable—is itself an objective of validation (§4.4). The intent is to demonstrate that the proposed constructs are measurable in principle, allowing the framework to be evaluated empirically rather than remaining purely descriptive.

## 4 Clinical utility, validation, and limitations

### 4.1 Prognostic stratification and longitudinal monitoring

If validated, stability classification may convey information beyond that available from symptom severity or resting laboratory measures (20–22, 39–41). Prognosis is position-dependent rather than diagnosis-dependent: patients with similar symptom burden or mediator profiles may occupy different regions of the fragility spectrum and follow divergent trajectories. Changes in recovery behavior, hysteresis, and synchronization may precede overt clinical deterioration or improvement, enabling earlier detection of destabilization and differentiation between transient fluctuation and meaningful changes in system behavior (18, 36–38).

**Figure 4:**
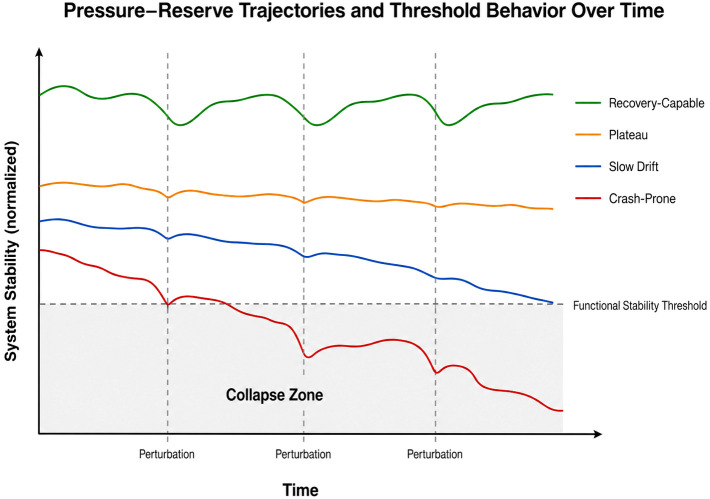
Pressure–reserve trajectories and threshold behavior over time. Longitudinal trajectory plot showing normalized system stability over time during repeated perturbations. The dashed horizontal line indicates the proposed functional stability threshold; the shaded region below represents the collapse zone. Recovery-capable (green): stability remains well above threshold with adaptive rebound following perturbation. Plateau (orange): stability remains above threshold but with a reduced reserve margin. Slow drift (blue): stability progressively erodes over time, approaching the functional stability threshold. Crash-prone (red): repeated threshold crossings are associated with prolonged recovery and incomplete restoration between perturbations. The figure illustrates the proposed threshold-crossing behavior that may distinguish crash-prone instability from gradual movement along the broader fragility spectrum.

Repeated assessment across the three stability axes enables trajectory tracking. Movement toward crash-prone behavior may be reflected in progressively prolonged recovery, increasing synchronization, and narrowing tolerance to perturbation; movement toward plateau and recovery-capable classifications is reflected in the reverse. This longitudinal perspective complements cross-sectional assessment and enhances interpretive depth. Pressure–reserve trajectories across the proposed classifications over time are shown in **Figure 4** (37, 38).

### 4.2 Interpretation of treatment variability

Treatment response in MCAS is often inconsistent and difficult to predict. Within this framework, such variability may reflect underlying differences in operating regime rather than intervention efficacy alone. Symptom improvement may reflect partial desynchronization, reduced perturbation amplitude, or temporary widening of functional margin without altering proximity to instability thresholds (20–22, 39).

An intervention that stabilizes symptoms in a recovery-capable or plateau classification may have limited or transient effect in individuals exhibiting slow-drift or crash-prone behavior, where perturbations propagate more readily across systems (40, 41). This framework does not prescribe treatment selection but provides a structured basis for interpreting why similar interventions yield divergent outcomes.

### 4.3 Research stratification and trial design

Clinical trials in MCAS frequently struggle with heterogeneous outcomes, as in clinical trials broadly (39–41). Stability-based stratification enables grouping of participants according to recovery behavior, reserve characteristics, and synchronization patterns rather than symptom count or mediator thresholds alone, reducing variance arising from unrecognized differences in system dynamics.

Potential applications include cohort enrichment by stability classification, stratified analyses distinguishing classification-specific response patterns, and improved interpretation of null or mixed results arising from unrecognized heterogeneity (39, 40). Future studies may also evaluate whether crash-prone behavior represents a reproducible threshold phenomenon distinct from gradual variation along the broader fragility spectrum.

### 4.4 Validation roadmap

Empirical validation should proceed in a staged sequence that evaluates the framework’s measurement structure before testing classification structure. A prospective observational design would enroll individuals meeting established MCAS criteria together with a healthy comparison cohort and combine questionnaire-derived stability measures with physiologic recovery assessment following a standardized submaximal perturbation. Candidate measures include heart-rate variability, symptom timing and duration, exertional and stress-exposure logs, sleep disruption, recovery trajectories, and optional biologic anchors such as lactate:pyruvate ratio (23, 27–29).

The first validation objective is construct validation of the proposed stability axes. Using the expanded research version of the State Classification Instrument (SCI), item-level psychometric analyses would determine whether Reserve Deficit, Reactive Pressure, and Synchronization emerge as separable constructs or collapse into a single severity dimension. Establishing axis independence represents the framework’s primary falsifiable prediction; failure to demonstrate separable dimensions would support reinterpretation of the model as a severity continuum rather than a multidimensional stability framework (36–38).

The second validation objective is determining whether participants form reproducible groupings within the three-axis space. Unsupervised approaches, including clustering and latent-class methods, would evaluate whether empirical classification structure emerges without imposing predefined categories. Recovery-capable, plateau, slow drift, and crash-prone classifications are therefore treated as reference hypotheses rather than assignment rules. If no reproducible groupings emerge, instability may be more appropriately modeled as a continuous fragility spectrum (37, 38).

The third validation objective is recovery correspondence. Data-derived classifications would be compared on physiologic recovery measures following standardized perturbation, including heart-rate recovery, heart-rate variability normalization, symptom trajectories, and lactate dynamics. The framework predicts that lower-reserve and higher-instability profiles will demonstrate slower and less complete recovery, prolonged recovery kinetics, and greater delayed symptom burden (12–14, 23, 29, 42).

A fourth objective is evaluation of threshold and hysteresis behavior. The central prediction of the framework is not the existence of four discrete classifications, but the existence of a reproducible crash-prone regime characterized by altered recovery dynamics. Candidate indicators include prolonged recovery, delayed worsening, increasing synchronization, paradoxical responses to throughput-increasing perturbations, and hysteresis, operationalized as recovery requiring a lower perturbation load than that associated with destabilization (20–22, 26). Because crash-prone behavior may involve risk of prolonged functional deterioration, validation of this regime should occur observationally through naturalistic 24–48-hour follow-up rather than deliberate provocation toward instability (12–14).

Finally, exploratory mechanistic anchoring may evaluate relationships between stability-axis scores and candidate bioenergetic markers such as lactate:pyruvate ratio. This measure is not required for validation of the framework itself but may provide biologic context for the Reserve Deficit and Reactive Pressure constructs (27, 28).

Because the framework is structured around falsifiable predictions, failure to demonstrate axis independence, reproducible classification structure, recovery correspondence, or threshold behavior would be considered informative outcomes that define the framework’s boundary conditions and guide subsequent revision rather than simple negative findings (36–38).

### 4.5 Illustrative clinical patterns

A crash-prone pattern is illustrated by delayed, multisystem instability following modest perturbation, analogous to post-exertional malaise described in related conditions (12–14). For example, moderate exertion may be followed hours later by clustered fatigue, cognitive dysfunction, gastrointestinal disturbance, and autonomic instability, with prolonged and incomplete recovery. Within the framework, this reflects constrained energetic reserve, dominant metabolic or exertional ingress, and high multisystem synchronization—consistent with proximity to functional thresholds and susceptibility to nonlinear collapse (20–22).

A defining feature of crash-prone behavior is not symptom severity alone, but altered recovery dynamics. Candidate manifestations include hysteresis, paradoxical worsening following attempts to increase activity, and failure to reliably return to baseline after perturbation. These features represent central targets for future validation.

In contrast, a recovery-capable pattern demonstrates transient, localized responses to similar perturbations without delayed or multisystem effects. Brief gastrointestinal or autonomic symptoms may occur following exertion but resolve rapidly without progression to fatigue, cognitive impairment, or cross-system involvement. This corresponds to energetic reserve exceeding reactive pressure, weak or context-dependent ingress, and low synchronization (20–22).

Plateau and slow-drift classifications are distinguished primarily by recovery dynamics and longitudinal trajectory rather than symptom type alone (42, 43). Plateau patterns demonstrate stable but constrained recovery with a limited functional margin; slow-drift patterns exhibit progressive lengthening of recovery time, increasing sensitivity to routine demands, and expanding multisystem involvement over time. Differentiation between these classifications emerges from how the system responds to load rather than from resting symptom burden (37, 38).

### 4.6 Limitations

The framework remains a conceptual and interpretive model. No empirical validation has been performed, no quantitative thresholds for the proposed stability axes have been established, and formal inter-rater reliability remains unquantified (36, 37). The proposed classifications should therefore be viewed as operational constructs requiring prospective validation rather than established biological categories.

Classification relies in part on patient-reported symptom timing, introducing potential recall bias. Several confounders warrant particular caution. Because reserve and synchronization are inferred from perturbation–recovery behavior, measurements are sensitive to factors unrelated to the proposed constructs: medications affecting heart rate variability, autonomic tone, or recovery kinetics; circadian timing, sleep quality, and recent activity or exertion; and acute intercurrent illness. Comorbidities common in this population—dysautonomia, connective-tissue disorders, and chronic fatigue or pain syndromes—may independently produce the same recovery and synchronization signatures the framework attributes to stability dynamics, making attribution difficult (1, 44). The endogenous, potentially feed-forward mast-cell pressure source described in §2.2 may also be difficult to disentangle from external or comorbid triggers. Robust validation will therefore require careful covariate control, repeated within-individual measurement, and comparison against appropriate non-MCAS reference samples. The proposed crash threshold remains hypothetical and may ultimately prove to represent a gradual transition rather than a discrete regime change.

The framework does not replace existing diagnostic criteria or biomarker-based approaches, but is intended to complement them by providing a structured means to interpret system behavior over time. Future studies will determine whether stability classifications, recovery dynamics, synchronization measures, and threshold behavior provide clinically meaningful information beyond existing diagnostic approaches.

## 5 Discussion

This paper proposes that MCAS is best understood as a disorder of state-dependent system instability rather than mediator burden alone (4–10). Clinical heterogeneity in MCAS—mediator–symptom mismatch, delayed crashes, inconsistent treatment responses—is reinterpreted here as reflecting differences in operating regime: patterns of stability and instability arising from the dynamic interaction of energetic reserve, reactive pressure, and multisystem synchronization, drawing on general systems models of instability and critical transitions (16–22). Within this framework, operating regimes offer a structured way to explain why individuals with similar mediator profiles may follow divergent trajectories, while avoiding any implication of diagnostic categorization or clinical assignment.

Importantly, the proposed classifications are not intended as severity grades. Individuals with similar mediator profiles, symptom burden, or laboratory findings may occupy different positions along the fragility spectrum and follow divergent trajectories, with differences emerging most clearly under perturbation rather than at rest (20–22, 39–41). Recovery-capable, plateau, and slow-drift classifications are best understood as operational regions along a continuum of increasing fragility rather than discrete biological categories. As summarized in **Table 3**, this distinguishes the framework from allostatic load, resilience indices, and severity staging: rather than summing cumulative burden or assigning an ordinal rank, it locates an individual on a continuous fragility spectrum and tests for threshold behavior.

**Table 3:** Comparison of the stability-based framework with existing systems-biology and resilience models.

Dimension	Allostatic load	Resilience indices	Severity staging	Stability-based framework (present)
Core unit	Cumulative physiologic burden	Capacity to resist/recover from stress	Ordinal disease severity	Position on a fragility spectrum; threshold behavior
Primary data	Aggregated/static biomarkers	Composite stress-response measures	Symptom/clinical thresholds	Perturbation–recovery behavior (kinetics, hysteresis, synchronization)
Shared assumption	Systems degrade as load accumulates	Stability depends on reserve/margin	Worse markers = worse disease	Stability depends on reserve relative to pressure
Key difference	Sums damage; not regime-based	General; not operationalized for MCAS	Static rank; ignores dynamics	Locates position; tests for a crash threshold
Advantage	Population-level burden	Intuitive, broad	Simple, familiar	Explains heterogeneity, delayed crashes, divergent trajectories
Limitation	Insensitive to current state	Hard to measure directly	No dynamics or mechanism	Unvalidated; thresholds not yet established

A central implication of the framework is that static biomarkers, however well validated, provide snapshots of system output at isolated time points rather than measures of the dynamic properties governing threshold proximity and stability. The same mediator level may reflect different functional realities depending on whether the system is operating with substantial reserve or exhibiting crash-prone behavior. Perturbation–recovery dynamics, by contrast, interrogate the system under load, revealing reserve capacity, failure propagation, synchronization, and recovery characteristics in ways that resting measurements cannot (18, 20–22, 26–28).

For MCAS specifically, this reorientation has practical significance. Mast-cell mediators influence neural, vascular, epithelial, immune, and autonomic systems simultaneously, allowing local perturbations to propagate across multiple domains. In this context, mediator release may function not only as a marker of activation but also as a contributor to reactive pressure and multisystem synchronization (7, 9–11). The framework therefore suggests that diagnostic and prognostic information may be more reliably extracted from how a system responds to challenge than from what it produces at rest—a principle consistent with established approaches in cardiology, exercise physiology, and systems neuroscience, where stress-testing rather than resting measurement has long served as a sensitive probe of functional reserve (12, 23, 29).

The framework builds upon established principles from nonlinear systems theory—including tipping points, hysteresis, critical slowing down, and state transitions—and maps them onto the multisystem physiology of MCAS (20–22, 26). Within this model, crash-prone behavior is proposed as a candidate threshold-crossing regime characterized by altered recovery dynamics, increasing synchronization, and impaired ability to re-establish baseline following perturbation. The central falsifiable prediction of the framework is therefore not the existence of four discrete states, but the existence of a reproducible crash boundary associated with measurable changes in system behavior.

This distinction has important implications for future validation efforts. Prospective studies should determine whether crash-prone individuals demonstrate hysteresis, paradoxical responses to throughput-increasing perturbations, prolonged recovery kinetics, and greater synchronization than individuals occupying other regions of the fragility spectrum. Validation of these features would support the existence of threshold behavior beyond what would be expected from a simple severity continuum.

More broadly, the approach may extend to other multisystem conditions characterized by threshold-dependent instability, including ME/CFS, fibromyalgia, dysautonomia syndromes, and related disorders in which delayed recovery, autonomic instability, exertional intolerance, and variable symptom propagation have been reported—features consistent with the pressure–reserve and synchronization dynamics proposed here (12–14, 24, 25, 42, 43, 45). In each case, similar stability-relevant features may be present; what may differ is the dominant pressure source, which in MCAS is the mast-cell mediator term described in §2.2. While MCAS serves as the model system examined here, the underlying principles may have broader applicability across conditions characterized by nonlinear instability and variable recovery behavior.

This work introduces a falsifiable stability-state classification framework linking perturbation–recovery dynamics to stability classification in MCAS. By shifting emphasis from mediator burden to operating regime, it provides a structured language for explaining heterogeneity, forecasting instability, and improving research reproducibility. This reflects a conceptual shift from mediator-centric assessment toward stability-state characterization grounded in system dynamics (20–22).

To understand instability, one must characterize the state of the system—not just the signals it emits.

## Data Availability

The original contributions presented in the study are included in the article/supplementary material. Further inquiries can be directed to the corresponding author.
